# MicroRNAs as Regulators of Insulin Signaling: Research Updates and Potential Therapeutic Perspectives in Type 2 Diabetes

**DOI:** 10.3390/ijms19123705

**Published:** 2018-11-22

**Authors:** Laura Nigi, Giuseppina Emanuela Grieco, Giuliana Ventriglia, Noemi Brusco, Francesca Mancarella, Caterina Formichi, Francesco Dotta, Guido Sebastiani

**Affiliations:** Diabetes Unit, Department of Medicine, Surgery and Neurosciences, University of Siena, Fondazione Umberto Di Mario ONLUS c/o Toscana Life Science, 53100 Siena, Italy; launigi@gmail.com (L.N.); giusy.grieco.90@gmail.com (G.E.G.); giulianaventriglia@gmail.com (G.V.); noemibrusco91@gmail.com (N.B.); francescamancarella90@gmail.com (F.M.); catefo@libero.it (C.F.); sebastianiguido@gmail.com (G.S.)

**Keywords:** microRNAs, insulin, insulin signaling, diabetes mellitus

## Abstract

The insulin signaling pathway is composed of a large number of molecules that positively or negatively modulate insulin specific signal transduction following its binding to the cognate receptor. Given the importance of the final effects of insulin signal transduction, it is conceivable that many regulators are needed in order to tightly control the metabolic or proliferative functional outputs. MicroRNAs (miRNAs) are small non-coding RNA molecules that negatively modulate gene expression through their specific binding within the 3′UTR sequence of messenger RNA (mRNA), thus causing mRNA decoy or translational inhibition. In the last decade, miRNAs have been addressed as pivotal cellular rheostats which control many fundamental signaling pathways, including insulin signal transduction. Several studies demonstrated that multiple alterations of miRNAs expression or function are relevant for the development of insulin resistance in type 2 diabetes (T2D); such alterations have been highlighted in multiple insulin target organs including liver, muscles, and adipose tissue. Indirectly, miRNAs have been identified as modulators of inflammation-derived insulin resistance, by controlling/tuning the activity of innate immune cells in insulin target tissues. Here, we review main findings on miRNA functions as modulators of insulin signaling in physiologic- or in T2D insulin resistance- status. Additionally, we report the latest hypotheses of prospective therapies involving miRNAs as potential targets for future drugs in T2D.

## 1. Introduction

Insulin resistance is a crucial feature and risk factor for Type 2 diabetes (T2D). Namely, it is defined as the failure of a given quantity of insulin to increase glucose uptake and utilization, and to facilitate survival and proliferation. Consequently, insulin resistance occurs primarily in the most insulin sensitive tissues such as liver, muscle and adipose tissue, but also in gastrointestinal tract [1], central nervous system [2], and in pancreatic β-cells [3]. It is relevant to mention that growing evidence suggests a relation between obesity, T2D, and brain disturbances [4]. Indeed, the defects of insulin signaling in classical peripheral target organs (liver, skeletal muscle and adipose tissue) are also associated with impaired insulin signaling in non-classical target organs (e.g., brain), which contribute to molecular and biochemical consequences of insulin resistance [5].

Insulin resistance is caused by several factors including obesity, inflammation, endoplasmic reticulum stress, and mitochondrial dysfunction. These factors differently contribute to the disruption of insulin signaling.

Insulin signaling is a tightly regulated pathway involved in the control of metabolic, pro-survival, and developmental cues in specific peripheral target tissues. Molecular defects in insulin signaling machinery components have been identified in insulin resistant status and in T2D), however a better knowledge on the mechanisms governing the activity of such signaling factors is still necessary. Although a detailed knowledge on some components, positively or negatively modulating insulin action, has been already reached, new classes of molecules are emerging as rheostats of multiple pathways and processes. Namely, microRNAs (miRNAs), small non-coding RNA molecules, represent new components of insulin signaling machinery capable of modulating the expression and activity of those factors actively involved in the regulation of insulin signal transduction. Importantly, miRNAs mediated insulin signaling modulation is tissue/cell restrained, with specific miRNAs modulating components of insulin transduction pathway only in some tissues or cells, thus rendering highly complex the understanding of these regulations.

The purpose of the present review is to provide an overview on the molecular basis of insulin signaling and to report an additional layer of regulation by mentioning the latest research updates on miRNAs in the regulation of insulin signal transduction components and their alterations in T2D.

## 2. Molecular Basis of Insulin and Insulin-Like Growth Factor I (IGF-1) Signaling

Insulin Receptor (INSR) downstream molecular signaling, is tightly regulated by a large number of factors and molecules, which contribute to a stringent control of energy homeostasis in peripheral insulin target tissues. Insulin signal transduction starts with its binding to INSR. It is a heterotetrameric tyrosine kinase receptor expressed on insulin sensitive tissues, composed by two extracellular subunits and two membrane/cytoplasmic subunits containing tyrosine kinase domains [6]. Two isoforms of INSR are known in mammals: isoform A, mainly expressed during fetal development and isoform B, expressed in mature insulin-sensitive tissues and mostly involved in insulin metabolic processes [7]. INSR is activated upon a specific conformational change in the cytoplasmic subunits induced by insulin binding to extracellular domains; such conformational change is followed by auto-phosphorylation of tyrosine residues [8], which represents an essential event for the recruitment of INSR downstream substrates [9]. The activation of INSR signaling necessitates the recruitment of phosphotyrosine-binding scaffold proteins such as SH2B1, SH2B2/APS, GRB10, GRB14 and/or GRB2. The best characterized scaffold molecules are those belonging to the Insulin Receptor Substrate (IRS) family, which bind INSR through their NH_2_-terminal pleckstrin homology (PH) and phosphotyrosine-binding (PTB) domains [6]. Although six IRS isoforms are known, the most functionally active in metabolic processes are IRS1 and IRS2. When phosphorylated, IRS proteins recruit phosphoinositide-3-kinase (PI3K) heterodimers, which contain a p85 (regulatory) and a p110 (catalytic) subunit. The master role of PI3K is to catalyze the production of phosphatidylinositol-3,4,5-trisphosphate (PIP3) from phosphatidylinositol-4,5-bisphosphate (PIP2), to allow the activation of a phosphorylation cascade of PH-domain containing proteins such as intermediate kinase effector Pyruvate Dehydrogenase Kinase 1 (PDK1) and PKB/Akt [10,11]. Additionally, PIP3 levels are controlled by specific phosphatases (e.g., PTEN, SHIP2) which control the reverse conversion of PIP3 to PIP2, thus providing a negative control on insulin signal transduction.

The insulin signaling central node, PKB/Akt, is activated upon its phosphorylation by PDK1 and/or mTOR complex 2 (mTORC2) [12]. Activated Akt is able to phosphorylate several specific substrates such as AS160, which promotes Glucose Transporter Type-4 (GLUT4) translocation and glucose uptake [13,14], Glycogen Synthase Kinase-3 (GSK-3), a serine/threonine kinase that inhibits glycogen synthase (GS) [15], and FOXOs, which modulate gluconeogenesis.

These components of insulin signaling pathway play pivotal roles through signaling machinery fine-tuning. Several defects in the activity of essential insulin signaling factors have been identified as responsible of insulin resistance in T2D pathogenesis.

Although several forms of severe insulin resistance syndrome, such as leprechaunism, Rabson-Mendenhall or type-A insulin resistance syndromes are characterized by INSR missense and/or non-sense mutations [16], these have not been observed in patients with T2D, indicating defects in others downstream insulin signaling components.

Indeed, in-vivo and in-vitro studies significantly highlighted the importance of insulin signaling molecules for proper functional effects of insulin. For instance, knockdown of Irs1 in L6 rat and in primary human myotubes, showed impaired insulin-stimulated glucose uptake [17,18,19]; similarly, liver specific Irs1 ablation leads to a marked glucose intolerance [20]. As a matter of fact, as demonstrated by Hribal ML and colleagues, G792R polymorphism of IRS1, which contributes to a decreased insulin sensitivity mediated by lower activation of PI3K, is more frequent in T2D patients vs. non diabetic controls [21]. Additionally, as demonstrated by several studies [3,22,23], systemic Irs2 knockout mouse models showed obesity and reduced insulin sensitivity, leading to impaired glucose tolerance and T2D; these results underline the importance of IRS scaffold proteins in insulin signal transduction.

The expression of PI3K has been shown to be reduced in type 2 diabetic skeletal muscle [24], and pharmacological PI3K inhibition abolished insulin stimulation of glucose transport and DNA synthesis. Indeed, PI3K subunits knockout models generally support the classification of PI3K as an additional essential node of insulin signaling [25,26,27,28,29].

Hepatic deletion of Akt1 and Akt2 isoforms resulted into glucose intolerance, insulin resistance and a defective insulin transcription in response to feeding [30]. Furthermore, knockdown or mutations of Akt2/PKBβ resulted into severe insulin resistance and altered glycemia, probably because defects of this important mediator lead to a reduction of AS160-induced GLUT4 translocation in both mice [31] and humans [32]. Equally important in insulin signaling pathway is the contribution of GSK-3. Overexpression of this factor in 293T and in 3T3-L1 cell lines leads to a reduction in GS affinity to glucose-6-phosphate and altered insulin-stimulated-IRS1 phosphorylation [33,34]. Furthermore, GSK-3 expression is increased in the skeletal muscle of type 2 diabetic patients and negatively correlated to whole-body insulin action [35]. 

Insulin resistance and diabetes have also been observed as a result of GLUT4 mutations or ablation. Heterozygous GLUT4+/− mice that display decreased GLUT4 protein in muscle and in adipose tissue are severely insulin resistant and predisposed to diabetes [36,37,38]. Interestingly, overexpression of GLUT4 in skeletal muscle of GLUT4+/− animals through crossing with transgenic mice, normalizes insulin sensitivity and glucose tolerance [39]. Transgenic mice expressing high levels of GLUT4 in adipose tissue or in skeletal muscle [40,41] are both highly insulin sensitive and glucose tolerant. Conversely, conditional depletion of GLUT4 in either adipose tissue or in skeletal muscle causes insulin resistance and diabetes [42,43].

The above reported molecular mechanisms triggered by insulin in target tissues, include some intermediate molecules which are shared by Insulin-like growth factor-I (IGF-1) signaling as well. IGF-1 has significant structural homology with insulin and is able to regulate insulin sensitivity in target tissues [44]. Indeed, IGF-1 and its receptor (IGF-1R) maintain insulin sensitivity in muscles [45]. Therefore, its role in glucose homeostasis should be considered. IGF-1 and insulin have different receptors whose different distribution allows their distinct but overlapping metabolic/pro-survival effects. Indeed, IGF-1 is involved in protein, fat and carbohydrate metabolism as well as proliferation and differentiation mainly through the activation of its receptor (IGF-1R) and the consecutive phosphorylation of tyrosine residues on the adaptor protein IRS-1 and IRS-2 [46]. This provides binding sites for the p85 subunit of PI3K, which is then activated.

IRS-1 and PI3K are essential for insulin signaling and action as well, thus suggesting that dysregulations of IGF-1 protein circulating levels may have a role in metabolic diseases. Of note, a specific polymorphism involving a tandem repeat in the IGF-1 promoter sequence is associated with an increased risk of development of type 1 diabetes [47]. Additional evidence showed that IGF-1 signaling is involved in the pathogenesis of T2D as well; IGF-1R knock-out of murine skeletal muscle cells induced progression toT2D over time [48]. In accordance, alterations of IGFBP expression levels has been observed in T2D and these changes result in free IGF-1alterations as well [49]. Moreover, in prediabetic patients, IGFBP-1 circulating levels are decreased, suggesting that this molecule is initially lower in subjects who will subsequently develop T2D, therefore representing a potential key molecule in metabolic control of glucose homeostasis.

Altogether, these data provide evidences that insulin signaling and/or IGF-1 signaling disruption at multiple levels may result into the alteration of insulin metabolic effects, thus contributing to insulin resistance and T2D. Therefore, a tight control, exerted by endogenous molecules positively or negatively engaged to modulate insulin signaling components, is strongly needed. Among several molecules and factors controlling the expression and/or activity of insulin or IGF-1 signaling components, miRNAs have been identified as potent modulators of multiple genes composing such complex molecular signaling machinery.

## 3. Regulatory Functions of miRNAs

MiRNAs are endogenous small non-coding RNAs (19–24 nucleotides) that negatively modulate gene expression by interacting with messenger RNA (mRNA) 3′-UnTranslated Region (UTR) of target gene, leading to mRNA degradation and/or translational repression. MiRNAs are involved in many cellular processes such as development, cell proliferation and survival, differentiation and apoptosis [50]. Their altered expression may contribute to several human diseases, including metabolic and cardiovascular disorders, cancer and neurological diseases [51].

Currently, 2588 annotated miRNA genes have been identified within the human genome. Importantly, approximately one third of protein coding genes are post-transcriptionally regulated by miRNAs, thus generating an additional layer of regulation [52,53].

miRNA genes are located within protein-coding genes or in independent non-coding RNA transcription units (TUs) [54]. Of note, most miRNA genes within protein-coding units (70%) are housed in intronic regions. However, both categories identified may exist in clusters consisting of multiple miRNA genes co-transcribed as poly-cistronic or as mono-cistronic primary transcripts [55].

Similarly to protein-coding genes, transcriptional regulation of miRNAs is an important step that determines their own expression and, therefore, activity. The identification of regulatory elements of miRNA genes at the DNA sequence level is obviously an important process to understand their regulatory mechanism. Some studies analyzed the genome context of human miRNA genes, providing a map of their enhancer/promoter regions and chromatin signature [56,57]. Moreover, miRNA promoter regions may contain CpG islands, TATA boxes, initiator elements, downstream promoter elements (DPE), TFIIB recognitions elements (BRE) and/or the proximal sequence elements (PSE) [58,59]. Collectively, these observations indicate that miRNA promoters can be regulated by transcription factors (TF), enhancer, silencing elements, and/or epigenetic modifications, thus being subjected to multiple external stimuli that determine their activity.

The canonical miRNAs biogenesis pathway starts in the nucleus (by RNA Polymerase II or RNA Polymerase III) to generate the long primary miRNA transcripts (pri-miRNA), which contain one or more hairpin structures [60,61]. The microprocessor complex composed of Drosha, an RNAse III enzyme, and its regulatory subunit DiGeorge Syndrome critical Region 8 (DGCR8), determines the cleavage of pri-miRNA to unfold the hairpin structured precursor (pre-miRNA). The precursor hairpin is then exported from nucleus to cytoplasm by Exportin-5 (Exp5) associated with Ran-GTP through nuclear pores. In the cytoplasm, Dicer, another RNAse III enzyme, processes the pre-miRNA by terminal-loop cleavage in order to generate a 21–24 nucleotide duplex miRNA. Finally, the main strand of the mature miRNA is loaded onto Argonaute (Ago) proteins to form the effector RNA-induced silencing complex (RISC).

MiRNAs drive RISC to specific gene transcripts to negatively regulate their expression by triggering mRNA degradation or translational repression [62,63], by binding to the complementary sequence in the 3′UTR of the specific targets [64,65]. MiRNAs can induce the mRNA decay through different molecular pathways. An initial step is represented by deadenylation through recruitment of deadenylases, which cause mRNA poly (A) shortening, oligo uridylation and mRNA decay [66,67,68]. Furthermore, RISC complex can recruit decapping factors onto the target mRNA removing the 5′ m^7^-cap structure, thus inducing mRNA degradation [69,70]. Finally, experimental evidences suggested that miRNAs can also positively modulate gene expression, by activating the translation with the same mechanisms previously mentioned or via non-canonical miRNA/mRNA interactions [71,72].

Such peculiar properties of miRNAs regulation of gene expression, generates a complex network of interactions in which a single miRNA can target multiple genes and, concomitantly, a single target gene can be regulated by multiple miRNAs, thus conferring them a powerful post-transcriptional expression control activity. Additionally, miRNAs gene expression control has been reported to be species- and cell-specific and importantly determined by cellular contexts, as recently reported [73]. Such feature, suggests the high complexity and flexibility of miRNAs regulations and their different effects depending on tissue/cell or species of interest.

## 4. Extracellular miRNAs

MiRNA specific regulatory activities can be exerted also in distant cells from those of origin. Indeed, increasing evidences highlighted that miRNAs can be secreted and detected in a circulating extracellular form. They are present in extracellular milieu in many different biological fluids including plasma/serum, urine, breast milk, aqueous humor. These miRNAs, released into body fluids can be involved in cell-cell communication. To exert this function, circulating miRNAs can be associated to small membrane vesicles (exosomes, microvesicles), lipoprotein complexes or carrier proteins (argonaute-2 proteins) [74,75]. Extracellular miRNAs can also be secreted by platelets, erythrocytes and all nucleated blood cells and, therefore, they might represent potential biomarkers for prediction and monitoring of several diseases including diabetes mellitus [76].

Given the complex scenario in the regulation of gene expression by miRNAs, it is clear that they can modulate entire pathways through the binding of specific target genes. Such activity can be undertaken at multiple levels, from the post-transcriptional regulation of receptor expression to final signaling effectors and transcription factors. Indeed, this is the case of insulin signaling components, which are regulated by several miRNAs whose altered expression has been detected in insulin resistance and in T2D.

## 5. MiRNAs as Rheostats of Insulin and IGF-1Signaling

The first report describing a role for miRNAs in the regulation of glucose homeostasis through the modulation of insulin signaling in peripheral target tissues, was published in 2006 and was referred to the function of a specific miRNA in *Drosophila Melanogaster* adipocytes [77]. Indeed, authors identified miRNA miR-278, prevalently expressed in Drosophila fat body adipocytes, and reported to target *Expanded* gene, a factor negatively involved in insulin signal transduction. As a matter of fact, miR-278-KO mutant flies, which showed higher expression of *Expanded* target gene in adipocytes, were hyperinsulinemic and hyperglycemic (in the form of trehalose), thus resembling the effect of insulin resistance. Despite higher insulin production, the authors specifically observed hyperactivation of FOXO in fat body adipocytes, a typical consequential effect of impaired insulin signaling and, therefore, of insulin resistant condition [78,79]. These findings highlighted the role of miR-278 in the modulation of insulin signaling in adipocytes, through the control of a specific target gene involved in insulin signal transduction.

**Figure 1:**
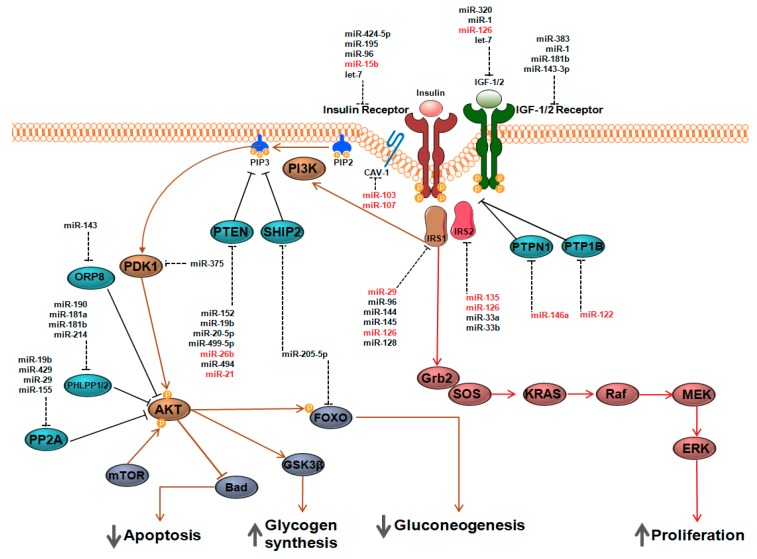
Expression of Insulin/IGF signaling components is modulated by miRNAs. Insulin Receptor (INSR) and Insulin-like Growth Factor (IGF) receptors are preferentially located into plasma membrane macrostructures (caveolae) characterized by caveolins proteins (e.g., Caveolin-1) and lipids, which stabilize their structures. Ligands binding to their specific receptors induces the activation of phosphatidylinositol 3-kinase (PI3K) and of other downstream components, mediated by the recruitment of scaffold elements (e.g., IRS1 and IRS2 protein members). PI3Kactivation, which triggers phosphorylation of PIP2 into PIP3, regulates insulin metabolic effects by phosphorylation of AKT through several intermediate kinases (e.g., PDK1), thus resulting into increased glucose uptake and glycogen synthesis and decreased gluconeogenesis. On the other side, insulin signal transduction induces a pro-survival status through the direct inhibition of Bad (pro-apoptotic) and activation of mitogen-activated protein kinase (MAPK) pathway (proliferation). MiRNAs negatively regulate multiple target genes involved in insulin/IGF signaling pathway and are indicated as dashed lines. MiRNAs targeting insulin signaling factors and identified as deregulated in insulin sensitive tissues in metabolic disease in man are depicted in red. The orange rounds with P in it represent phosphate groups; the grey arrows pointing down indicate negative regulation of those specific process and the grey arrows pointing up indicate positive regulation of those specific process. Factors reported in brown represent the main effectors of insulin signaling metabolic activations; factors reported in dark red represent the activated effectors acting on proliferation and pro-survival cues; factors reported in light blue represent phosphatases and kinases; factors reported in dark blue represent interactors /effectors of Akt signaling. Note: PTEN (Phosphatase And Tensin Homolog), GAV (Gill-associated Virus), PTPN1 Protein Tyrosine Phospatase, Non-receptor type 1, PTP1B Protein-tyrosine phosphatase 1B, PDK1 (Phosphoinositide-dependent kinase 1), ORP8 (OSBP-related protein 8), PHLPP1/2 (Pleckstrin Homology Domain Leucine-rich Repeat Protein Phosphatase1/2), PP2A (Protein Phosphatase 2 A), AKT (Protein Kinase B), mTOR (mammalian Target of Rapamicin), GSK3β (Glycogen Synthase Kinase 3 β), FOXO (Forkhead box O), SOS (Son of Sevenless), KRAS (Ki-ras2 Kirsten rat sarcoma viral oncogene homolog), MEK (Mitogen-activated protein kinase kinase), ERK (Extracellular Signal-Regulated Kinase).

Such initial results paved the way to a broader role for miRNAs in the regulation of peripheral tissues insulin sensitivity, thus pushing more research studies to further explore this field. Indeed, at present, strong evidences indicate that miRNAs are deeply involved in the post-transcriptional fine tuning of several insulin signaling components, from insulin receptor (INSR) to transcription factors; of note, some are altered in T2D and in insulin resistance (Figure 1). Additionally, several other studies highlighted the importance of miRNAs in the regulation of IGF-1 signaling, thus suggesting that miRNAs are involved in the regulation of insulin sensitivity and/or proliferation/differentiation cues through the modulation of IGF-1 signaling as well.

### 5.1. Insulin Receptor (INSR)

A recent report demonstrated the direct interaction of miR-424-5p with INSR mRNA 3′UTR sequence in human hepatocytes cell line HepG2 [80], showing that overexpression of miR-424-5p induced a reduction of INSR mRNA and protein levels. Additionally, it provided evidence that HepG2 cells treated with saturated fatty acids, as well as liver from high fat diet (HFD) mice, retained a reduced expression of INSR and IRS1 paralleled by an increased expression of miRNA miR-424-5p. These data suggest that miR-424-5p hyperexpression was potentially driven by an acute lipotoxic stress and lipid accumulation leading to downregulation of INSR. Although the transcriptional activation mechanism and expression regulation of miR-424-5p is largely unknown, it is likely that its hyperexpression upon fatty acids treatment can be partly attributed to the activation of TGFβ-SMAD3 signaling [81], suggested by the presence of several SMAD3 binding sites within miR-424-5p human promoter region [80].

INSR has been previously reported to be the target of miRNA miR-15b. In liver of Diet Induced Obesity (DIO) mice, miR-15b levels were increased and paralleled by decreased expression of INSR, both at mRNA and at protein level. The direct relationship between miR-15b and INSR was established by 3′UTR-luciferase reporter assay and by showing the decreased expression of INSR upon miR-15b overexpression in HepG2 cell line, thus demonstrating that such miRNA may play a role in the regulation of insulin signaling in mouse and human hepatocytes [82]. Of note, miR-15b and miR-15a expression has been recently found increased in skeletal muscle biopsies obtained from offspring of women affected by gestational diabetes mellitus (GDM) vs. offspring from non-diabetic women during pregnancy. As a matter of fact, GDM women offspring exhibit insulin resistance and increased risk to develop T2D later in life [83]. These data are in line with the previous report showing an association between miR-15b and insulin resistance development [82].

Regarding other miRNAs targeting INSR, it has been reported that saturated fatty acids (SFAs) increased miR-195 and miR-96 expression in HepG2 cells; such miRNAs were both reported to directly target INSR, whose expression was reduced in HepG2 upon SFAs treatment and in liver from HFD mice [84,85].

INSR signal transduction and efficient downstream activation is also partially dependent on plasma membrane macro-structural motifs determined by caveolins proteins. For instance, Caveolin-1 (Cav-1), a member of caveolin proteins family composing caveolae structures, is essential for INSR compartmentalization and multiple signals integration, stabilizing INSR structure for efficient insulin signaling. It has been demonstrated that miR-103 and miR-107 directly bind Cav-1 3′UTR sequence, thus controlling its expression. These miRNAs were found upregulated in liver from ob/ob and DIO mice and reportedly contributing to insulin resistance, resulting in increased hepatic glucose production through augmented gluconeogenesis. The role of miR-103/107 has been also explored in adipocytes of ob/ob and DIO mice, where miRNAs in-vivo silencing (using antagomiRs) generated the major effects in terms of normalization of glycemia and improved glucose homeostasis respect to their silencing in the liver. However, both in fat and liver of ob/ob and DIO mice, the inhibition of miR-103 alongside with miR-107, increased Cav-1 levels and contributed to INSRβ-subunit hyperexpression and enhanced AKT activity (characterized by a higher proportion of p-AKT detected) [86]. Importantly, a similar miR-103/107 expression pattern has been detected in liver of patients suffering from NAFLD and NASH, both conditions potentially associated with diabetes and insulin resistance [86].

### 5.2. Protein Tyrosine Phosphatases (PTPAse)

Other downstream insulin signaling components, directly or indirectly interacting with INSR phosphorylated tyrosine residues and controlled by specific miRNAs, have also been widely explored and identified. This is the case of protein tyrosine phosphatases (PTPAses), which negatively modulate insulin signaling by removing phosphate groups from tyrosine residues of the cytoplasmic domain of INSR. One of them, namelyPTPN1, has been reported to be a predicted target of miR-146a; indeed, PTPN1 expression was inversely correlated with miR-146a both in skeletal muscle and in liver of a T2D rat model. As a matter of fact, while PTPN1 expression was found upregulated, miR-146a was downregulated, thus linking miR-146a to the modulation of PTPN1 expression [87]. Of note, miR-146a has been widely investigated in human T2D pathogenesis and several findings reported its downregulation in whole blood, in plasma and in some peripheral tissues [88]. Another miRNA, miR-122, a widely evolutionary conserved miRNA highly enriched in hepatocytes, has been reported to directly target a different member of PTPases family: PTP1B. Liver-specific deletion of PTP1B in HFD mice, led to the improvement of glucose homeostasis (enhanced INSR phosphorylation, increased insulin sensitivity), thus implying a negative role, as expected, for PTP1B in insulin sensitivity [89]. On the contrary, wild type HFD mice hepatocytes showed a significant miR-122 downregulation, driven by the reduced expression levels of its liver-specific transcription factor HNF4α, leading to PTP1B hyperexpression and insulin signaling impairment due to low levels of phosphorylation at specific tyrosine residues [90].

### 5.3. Insulin Receptor Substrates (IRS)

The best characterized molecules downstream of the INSR, upon its cytoplasmic domain phosphorylation, are IRS proteins. The main IRS members, i.e., IRS1 and IRS2, have been demonstrated to be targeted by several miRNAs in multiple peripheral insulin target tissues. For example, miR-222 has been shown to be upregulated in mouse liver upon high fat/high sucrose diet +gold thioglucose; in the same study, authors showed that in primary mouse hepatocytes, miR-222 directly targets IRS1 thus inducing insulin resistance [91]. This miRNA was also found hyperexpressed in adipocytes from HFD mice and its function has been associated to adipogenesis in mouse and human context as well [92,93]; we may therefore speculate that increased expression of miR-222 may dampen insulin signaling in adipocytes by targeting IRS1 as well as others downstream effectors, thus contributing to insulin resistance also in adipocytes.

In human hepatocyte cell line SK-Hep1, IRS1 has been reported to be a direct target of miR-126 and miR-96. Increased miR-126 and miR-96 expression and parallel IRS1 downregulation were consequent to mitochondrial-induced dysfunction, which caused impaired insulin signaling and reduced glycogen synthesis [94,95].

IRS1 expression levels in hepatocytes have been reported to be modulated also by miR-145 [96]; such miRNA was found upregulated in liver of mice treated with resistin, thus being involved in resistin-induced insulin resistance through IRS1 expression levels reduction [97].

In other insulin target tissues (e.g., skeletal muscle), IRS1 has been reported to be regulated by other miRNAs such as miR-128a [98]. Such regulatory mechanism has been ascribed to those processes contributing to the regulation of myoblasts proliferation and muscle regeneration during myogenesis; although not directly proven to be altered in insulin resistance condition, miR-128a has been demonstrated to be negatively controlled by TNFα, a cytokine contributing to inflammation in T2D. 

Another miRNA targeting IRS1 in myocytes (L6 GLUT4myc cell line derived from rat skeletal muscle) is miR-29a. Indeed, miR-29a directly targets IRS1 3′UTR, thus repressing its expression; moreover, miR-29a is induced upon saturated fatty acid (palmitate) treatment, therefore contributing to the development of insulin resistance [99]. Importantly, miR-29a and miR-29c (but not miR-29b) resulted hyperexpressed in skeletal muscle biopsies from T2D patients vs. normal glucose tolerant (NGT) subjects [100]. Accordingly, overexpression of miR-29a and miR-29c in cultured human skeletal muscle primary cells impaired glucose metabolism by altering glucose uptake and by impairing insulin signaling through the reduction of hexokinase 2 (HK2), GLUT1, IRS1 (as previously demonstrated), PIK3R3 and AKT2 mRNA and protein expression. Conversely, inhibition of miR-29a and miR-29c in human myotubes ameliorated and improved glucose metabolism, thus underlining a pivotal role for miR-29 miRNAs as modulators of glucose utilization in skeletal muscle, through insulin signaling regulation [100].

Finally, IRS1 was also demonstrated to be a target gene of miR-126a in mouse endothelial cells, involved in the control of cell viability and proliferation; specifically, miR-126a was found downregulated in retinal endothelial cells from a mouse model of diabetic retinopathy and contributed, through IRS1 modulation, to dysfunctional angiogenesis [101].

MicroRNA miR-126 has been found to target IRS-2 as well [102]; in this study the authors found that miR-126 hyperexpression in INS-1β cell line (rat β-cell line derived from insulinoma) induced a reduction of proliferation via downregulation of IRS-1 and IRS-2. Such reduction was restored only when IRS-2 was overexpressed while no effects were observed for IRS-1, thus suggesting that in β-cells miR-126 modulates proliferation preferentially through the regulation of IRS-2. Additional miRNAs have been demonstrated to control the initial steps of insulin signaling by targeting IRS2. In hepatocytes miR-33a and miR-33b, identified as intragenic miRNAs located within the human sterol regulatory element-binding protein-2 and -1 genes (SREBP-1, SREBP-2), have been reported to modulate fatty acid and cholesterol metabolism, as well as insulin signaling, by targeting IRS2 [103,104]; accordingly, miR-33b overexpression in Huh7 human hepatocytes cell line resulted into AKT and ERK reduced phosphorylation secondary to IRS2 down-regulation. In muscles, IRS2 has been also shown to be targeted by miR-135a, whose overexpression in C2C12 cells (immortalized mouse myoblast cell line) impaired insulin signaling by via IRS2 down-regulation. Furthermore, miR-135a was increased in muscle biopsies obtained from T2D patients and in gastrocnemius muscle of db/db mice. Importantly, in-vivo inhibition of miR-135a in db/db mice, a T2D mouse model characterized by muscle miR-135a hyperexpression, ameliorated glucose metabolism and improved glucose tolerance through the restoration of IRS2 and of p-AKT expression levels in gastrocnemius skeletal muscle [105].

### 5.4. PTEN and SHIP2

As mentioned above, IRS proteins link INSR activation to insulin metabolic effects through the intermediate modulation of phosphatidylinositol 3-kinase (PI3K)/PDK1/AKT pathway. PI3K activity, which determines PIP3 production, is regulated by PTEN and SHIP2 which, in turn, dephosphorylates PIP3, thus reducing the availability of the final effector of PI3K signal transmission. Expression and activity of PTEN and SHIP2 has been demonstrated to be tightly controlled by several miRNAs. In hepatocytes, glycogen synthesis mediated by insulin signaling has been widely associated with miRNA-PTEN regulatory networks; indeed, it has been demonstrated that miR-152 [106], miR-19 [107], miR-20-5p [108], and miR-499-5p [109] directly target PTEN phosphatase and were reported to be downregulated in liver from T2D mouse models (e.g., db/db mice), paralleled by PTEN hyperexpression and signs of hepatic insulin resistance. PTEN hyperexpression, secondary to down-regulation of above mentioned miRNAs, resulted into insulin signaling impairment through a significant reduction of AKT activity and, consequently, of downstream effectors.

In skeletal muscles, PTEN expression is modulated by other miRNAs such as miR-21 [110] and miR-494 [111]; as for miR-21, accumulating evidence showed and confirmed its association with PTEN post-transcriptional regulation in multiple cellular contexts (adipocytes, hepatocytes, and myotubes), thus finally attributing a pivotal role to this miRNA in the modulation of insulin signaling through the regulation of PTEN phosphatase in a wider set of tissues [112,113]. Despite the strong evidence of miR-21 expression levels alteration in T2D mouse models and of its involvement in the regulation of insulin signaling, no direct confirmation of altered miR-21 expression in human T2D insulin target tissues has been provided yet; although, altered circulating miR-21 levels have been reported in plasma/serum of T2D patients and of obese subjects [114,115].

In another study, a miRNA expression profile analysis in visceral adipose tissue from a rodent model of obesity and from obese subjects revealed a decreased expression of miR-26b. This miRNA was reported to target PTEN, thus leading to its hyperexpression when miR-26b levels were reduced. In physiologic conditions, normal levels of miR-26b are needed in order to control PTEN expression/activity, thus allowing insulin signal transduction, GLUT4 translocation and appropriate glucose uptake in human adipocytes. In the same study, the authors demonstrated multiple effects of miR-26b on glucose homeostasis by showing its role in the regulation of pancreatic islets function as well; indeed, a significant downregulation of miR-26b was found in islets and in serum of HFD mice. Furthermore, it was demonstrated that miR-26b hyperexpression protected mice from HFD induced diabetes, possibly mediated by improved β-cell function and by restoring the expression levels of its target genes (e.g., PTEN) in adipocytes [116].

Conversion of PIP3 into PIP2, involved in the negative modulation of AKT activity through depletion of PIP3 levels, is mediated by SHIP2. A recent report [111] showed that this phosphatase is targeted by miR-205-5p, which regulates its expression levels. In this study, it was demonstrated that hepatocytes from FOXO-KO mice show an altered miRNAs expression profile, leading to the hypothesis that FOXOs regulate miRNAs expression by directly controlling them at transcriptional levels or via other indirect mechanisms. Among these miRNAs, miR-205-5p was identified as the most upregulated miRNA in hepatocytes from FOXOs-KO mice and found to directly inhibit SHIP2. Additionally, a direct post-transcriptional control of FOXO1 by miR-205-5p was detected, thus suggesting a regulatory loop capable of modulating insulin signaling through the regulation of SHIP2 and FOXO1. Indeed, in-vitro overexpression of miR-205-5p in primary human hepatocytes activated AKT signaling as shown by reduction of SHIP2, downregulation of FOXO1, upregulation of p-FOXO1 and increased activity of AKT through its increased phosphorylation. As a result of insulin signaling activation, miR-205-5p overexpression led to a decrease in hepatic glucose production. As a matter of fact, in conditions of insulin resistance, miR-205-5p was found downregulated in hepatocytes from db/db mice and in a limited cohort of T2D patients, therefore underlining the importance of miR-205-5p in the regulation of glucose homeostasis both in physiologic and in pathologic conditions [117].

Besides its indirect effects through the modulation of miRNAs targeting phospholipid phosphatases PTEN and SHIP2, PI3K has been also reported to be directly targeted by miRNA miR-128a, which negatively modulates phosphatidylinositol 3-kinases regulatory 1(PIK3R1) gene [98,118], further reinforcing the role of miRNAs in the regulation of the early steps of insulin signaling.

### 5.5. AKT Activity Modulators

The central node of insulin signal transduction is represented by AKT activation or inhibition, which is mediated by specific kinases or phosphatases.

PDK1 is an intermediate kinase activated by PIP3, thus resulting in the final activation of AKT. PDK1 has been reported to be targeted by several miRNAs. The first miRNA identified as regulator of PDK1 expression is miR-375. The role of miR-375 as PDK1 expression regulator has been mainly investigated in β-cells, where its overexpression led to a reduction of PDK1 mRNA and protein levels [119]. Hyperexpression of miR-375 and downregulation of PDK1, induced a significant decrease of glucose-induced insulin secretion. In INS1-E and primary rat islets, glucose itself induced a decrease of miR-375 expression levels and a concomitant increase of PDK1 protein expression, providing evidence for a role of miR-375 in the regulation of PDK1. However, although no direct evidence of a role of miR-375 in the regulation insulin sensitivity is at present available, the modulation of miR-375expression could represent a central hub for the regulation of insulin resistance in peripheral tissues as well [120]. AKT has also been recently reported to be indirectly regulated by miR-143 trough the modulation of Oxysterol binding protein related 8 (ORP8). AKT is directly regulated by (ORP8), and specific silencing of this gene significantly decrease insulin-AKT signaling pathway [121,122]. Kitamura and colleagues confirmed the targeting of miR-143 on ORP8 3′UTR [123], thus suggesting that insulin-AKT signaling could be impaired by miR-143 expression level alterations mainly through the downregulation of ORP8.

Additional AKT-downstream kinases and phosphatases acting on specific aminoacidic residues and determining activation or inhibition of AKT activity itself, represent major regulators of the entire insulin signaling. Protein phosphatase 2a (PP2A) activity, responsible for direct inactivation of AKT, has been reported to be lost in many human cancers, thus leading to loss of control of proliferative output determined by insulin signaling. PP2A activity was found increased in primary rat hepatocytes in insulin resistance induced by palmitate treatment. Moreover, PP2A mRNA was increased in liver, muscle and adipose tissue derived from insulin resistant Zucker Diabetic Fatty (ZDF) rats, thus suggesting a role for PP2A in the deregulation of insulin signaling in T2D. Importantly, PP2A expression is modulated by several miRNAs including miR-19b [124], miR-429 [125], miR-29 [126] and miR-155 [127], whose expression was in turn found altered in diabetes. Other phosphatases directly involved in AKT inactivation are the Pleckstrin Homology domain leucine-rich repeat protein phosphatases-1 and -2 (PHLPP1, PHLPP2). In consequence of elevated expression levels of PHLPPs reported in insulin target tissues (subcutaneous adipose tissue, skeletal muscle, myotubes) of T2D and obese patients [128,129], and experimentally associated to an insulin resistance status, PHLPPs have been considered as potential therapeutic targets of for insulin resistance treatment [130]. This requires a detailed understanding of PHLPPs regulation including that exerted by miRNAs; indeed, miR-190, miR-214, miR-181a and miR-181b have been demonstrated to target PHLPP1 and PHLPP2 [131,132,133]. Particularly, miR-181b expression was found reduced in adipose tissue vasculature of obese mice, thus leading to an increased expression of PHLPP2 and inactivation of Akt, which resulted into impaired insulin signaling. Rescuing miR-181b expression by delivery of its mimic, improved glucose tolerance through enhanced insulin-mediated AKT phosphorylation at Ser473, and was associated to reduced endothelial cell activation and decreased inflammation, with a shifted macrophage polarization towards a M2 anti-inflammatory state in the adipose tissue [134]. These effects were associated with an induction of endothelial nitric oxide synthase, nitric oxide activity and FoxO1 phosphorylation specifically in epididymal white adipose tissue, but not in liver nor in skeletal muscle. As previously indicated, bioinformatic and gene expression profile studies revealed that PHLPP2 is a novel target of miR-181b. Indeed, in-vivo knock down of PHLPP2 expression recapitulated miR-181b protective effects on glucose homeostasis, insulin sensitivity and inflammation in epididymal white adipose tissue. Thus, these findings highlight how miR-181b plays an important homeostatic role by controlling visceral fat endothelial cell inflammation and insulin resistance [134]. In addition to these mechanisms exerted by miRNAs during inflammatory conditions, inflammation can lead to decreased insulin action also through the direct modulation of PI3K pathway. Indeed pro-inflammatory cytokines can activate downstream kinases of insulin signaling pathway, including IκB kinase-β (IKKβ), JUN amino-terminal kinase 1 (JNK1) and p38 MAPK, which can contribute to increased serine phosphorylation that inactivates IRS protein and stimulate the production of suppressors of cytokine signaling (SOCS), well known negative regulators of insulin signaling, linking insulin resistance, inflammation and cytokine signaling [135].

### 5.6. Insulin-Like Growth Factor-1 (IGF-1) and Insulin-Like Growth Factor-1 Receptor (IGF-1R)

The regulation of IGF-1 expression and secretion as well as the modulation of its receptor (IGF-1R), has been reported to be controlled by miRNAs as well.

miR-320 has been reported to modulate IGF-1 expression in3T3-L1 adipocytes. Of note, it is also able to control insulin sensitivity in 3T3-L1 adipocytes through insulin signaling pathway modulation [136], thus highlighting the overlapping functions of some miRNAs in the regulation of insulin and/or IGF-1 signaling pathways. Another important miRNA involved in IGF-1 signaling is miR-126, which is downregulated in proliferative diabetic retinopathy and involved in neovascularization. This condition is mediated by several angiogenic factors such as VEGF, HIF-Iα and IGF-1 and IGF-2. Most importantly, it has been demonstrated that the restoration of miR-126 expression levels is able to increase the expression of essential angiogenic factors including IGF-1 and IGF-2, thereby ascribing miR-126 to the plethora of miRNAs regulating IGF expression and signaling in target cells [137]. In support of this, Rivas et al. used miR-126 gain- and loss-of-function experiments in skeletal muscle cells to demonstrate that it functionally regulates signal transduction in response to IGF-1 treatment, by modulating Akt signaling [138].

Additionally, miR-126 has been found dysregulated in T2D and in diabetic complications in multiple tissues [139,140]; these evidences highlighted the importance of miR-126 in the regulation of IGF signaling also in T2D.

Additional evidences suggested such overlapping mode of regulation; for example, let-7 directly binds the 3′UTR of IGF-1R, IRS-2 and INSR mRNA thus indicating that let-7 may function as a regulator of glucose metabolism by targeting multiple components of growth/metabolic signaling pathway genes including IGF-R as well [141]. Of note, Zhu and colleagues demonstrated that let-7 transgenic mice showed impaired glucose tolerance, thus confirming the multilayer regulatory role of let-7 [141].

IGF-1R has been reported to be targeted by miR-214 as well. Indeed, it has been recently reported to directly bind the 3′UTR of IGF-1R and to negatively modulate its mRNA as well as protein expression in ACHN, 786-O, A498, and RCC4 renal cancer cells. Moreover, miR-214 is able to regulate IGF-1-stimulated Akt kinase activity in renal carcinoma cells [142]; of note, as previously reported, miR-214 has been tightly associated with insulin resistance in endothelial cells [133] and in muscle cell lines alongside with miR-135 and miR-202, thus underlining the potential role of miR-214 to contribute to insulin resistance through multiple networks regulation as previously reported for let-7 [133,143].

Other authors demonstrated that miR-1 regulates the expression of both IGF-1 and IGF-1R in cardiac and skeletal muscles [144]; additionally, in this report, the authors demonstrated that miR-1 expression levels are reduced and inversely correlated to IGF-1 expression in a cardiac mouse model of hypertrophy [144].

The contribution of IGF signaling in T2D and metabolic diseases has been further elucidated in a recent report showing the differential expression of miR-143-3p in serum of patients with metabolic syndrome. Indeed, this miRNAs was found significantly hyperexpressed in serum of these patients as well as in the serum of HFD mice. Importantly, miR-143-3p hyperexpression was also detected in heart, pancreas, and skeletal muscle of the same set of obese mice, thus defining the origin of circulating miRNA alteration. miR-143-3p has been found to target IGF-2R receptor and IGFBP5 thus potentially contributing to insulin resistance observed during metabolic syndrome [145].

Since IGF signaling pathway has been addressed as highly involved in cancer cells growth, some reports linked IGF signaling to specific miRNAs regulation in several tumor cells. As an example, in glioma, the downregulation of miR-383 stimulates AKT signaling and controls the expression of Matrix Metalloproteinase II (MMP2), through the regulation of IGF-1R, thereby controlling tumor cells invasiveness [146]. In the same type of cancer, it has been additionally demonstrated that miR-181b directly modulates IGF-1R. Indeed miR-181b in-vitro overexpression reduced IGF-1R expression levels; in glioma clinical specimens, an inverse correlation between miR-181b and IGF-1R has been observed thus confirming the in-vitro results [147]. Importantly, as reported above, miR-181b has been associated to the modulation of insulin resistance in adipose tissue. Although in a different context, miR-181b may regulate IGF signaling in adipose tissue as well. Therefore, additional studies are required to better characterize miRNA-mediated regulation of insulin and IGF signaling.

Additional layers of regulation has been reported for many other microRNAs, whose differential expression promotes cell proliferation, migration, invasion, and tumorigenesis by targeting IGF-1R and the signaling pathways-PI3K/AKT and MAPK/ERK1/2 as extensively reviewed here by others [148].

## 6. Extracellular miRNAs as Regulators of Insulin Sensitivity

Two breakthrough studies have recently shown how circulating exosomal miRNAs released into extracellular space can modulate intercellular communications, thus influencing metabolic homeostasis. Indeed, both adipocytes and macrophages derived from adipose tissue can release exosomal vesicles containing miRNAs to exert distant metabolic function. For instance, Ying et al. have examined exosomes of macrophages derived from adipose tissue collected from obese and lean mice. They found that treatment of lean recipient mice with macrophages exosomes derived from adipose tissue collected from obese mice led to transfer of miRNAs to insulin sensitive tissues (muscle, liver, and adipose tissue) leading to impairment of glucose tolerance and enhancing insulin resistance. Conversely, treating obese insulin resistant mice with adipose tissue derived exosomes of lean mice greatly improved glucose tolerance and insulin sensitivity [149]. Moreover, authors have identified miR-155 as one of miRNAs overrepresented in exosomes of adipose tissue derived macrophages from obese mice, suggesting a potential function of miR-155 through the regulation of PPARγ, a known miR-155 target gene. Indeed, miR-155 KO mice fed with high fat diet were insulin sensitive and glucose tolerant compared to control mice. Accordingly, transplantation of wild-type bone marrow into miR-155 KO mice mitigated the insulin sensitive/glucose tolerant phenotype [149].

Another breakthrough study was published by Thomou and colleagues, who aimed at establishing whether exosomal miRNAs released by adipose tissue could regulate gene expression in distant organs [150]. They firstly observed that adipose tissue contributes greatly to the enrichment of circulating exosomal miRNAs; indeed, mice lacking Dicer specifically in adipocytes showed a sharp reduction in the level of circulating exosomal miRNAs. A similar decrease was also found in patients suffering from a generalized loss of adipose tissue. In the mouse model, the level of circulating exosomal miRNAs could be partially restored by transplanting different fat depots from wild type mice, especially brown adipose tissue, which improved insulin sensitivity. Gaining insight into one of the possible mechanisms underlying the link between circulating exosomal miRNA and insulin sensitivity, it has been shown that exosomal miR-99b from the adipose tissue could be uptaken by the liver, in which it restrains the expression of hepatic fibroblast grow factor 21 (FGF21), resulting into a glucose intolerant phenotype [150]. Taken collectively, these two studies highlight how adipose tissue in physiological or inflammatory conditions can influence distant insulin sensitive tissues through miRNAs, thus adding additional layers of complexity such as miRNA mediated organ cross-talk to insulin signaling, insulin sensitivity and glucose homeostasis.

## 7. Future Therapeutic Perspectives

As mentioned above, a role for miRNAs in the regulation of glucose homeostasis, through the modulation of insulin signaling in peripheral target tissues, has been widely reported in literature. Indeed, from the first observation by Teleman et al. [77], many studies have shown a direct involvement of miRNAs in β-cell dysfunction and insulin resistance. Such a key role in regulating the expression of significant protein cascades of insulin signaling pathway affecting insulin resistance and T2D, makes miRNAs attractive therapeutic targets. Increasing evidence in animal models suggests that strategies aimed at modulating miRNA expression in-vivo, could have a positive impact on the treatment of diabetes as well as of insulin-resistance [151,152].

To date, two different therapeutic approaches have been developed: (*i*) restoration of expression of those miRNAs that are downregulated in the diseased state, by either synthetic double-stranded miRNAs (miRNA mimics) or viral expression vector; (*ii*) inhibition of miRNAs activity by either antisense oligonucleotides (anti-miRNAs) or competitive miRNA inhibitors (miRNA sponges). It is obvious that, depending on the role of the specific miRNA, the goal of the treatment could be to either increase or diminish its function. At present, anti-miRNAs are the most common approach in-vivo in designing miRNA-based therapies [152,153].

In T2D and in insulin-resistance, some pioneering studies reported the potential in-vivo application of miRNAs inhibition for therapeutic purpose [86]. As previously mentioned, Trajkovski et al. observed that inhibition of miR-103 and miR-107 by antisense oligonucleotides in liver and in adipose tissue of DIO and ob/ob mice improved glucose homeostasis and insulin sensitivity. In contrast, injections in liver of an adenoviral vector expressing miR-107 resulted into elevated fasting blood glucose and insulin levels, impaired glucose tolerance after an i.p. glucose injection, decreased insulin sensitivity and increased hepatic glucose production. Of note, a GalNAc-conjugated oligonucleotide targeting miR-103/107 (RG-125, AZD4076) is currently in a phase I/IIa clinical trial evaluating safety and tolerability of AZD4076 and assessing its effect on insulin sensitivity and liver fat content in patients with T2D and non-alcoholic fatty liver disease (NAFLD) [154]. Esau et al. demonstrated the potential therapeutic effects of liver-specific miR-122 inhibition in DIO mouse model by using antisense oligonucleotides [155]. Indeed, inhibition of miR-122 induced an improvement in liver steatosis and a reduction of plasma cholesterol levels; accordingly, a suppression of several lipogenic genes was observed following this strategy. These results suggest a role of miR-122 as a therapeutic target for metabolic diseases. Subsequently, Zhou et al. showed that a specific downregulation of miR-181a (with an antisense oligonucleotide) improved hepatic insulin sensitivity and glucose homeostasis in DIO mice and increased SIRT1 protein levels and activity. As a matter of fact, miR-181a was shown to be a miRNA targeting SIRT1. Conversely, overexpression of miR-181a caused impaired hepatic insulin signaling and decreased SIRT1 protein levels and activity [156].

Finally, Seeger et al. suppressed miR-21 expression in white adipose tissue of db/db mice by using locked nucleic acid-modified anti-miR (LNA-21). The inhibition of miR-21 resulted in a reduction of body weight, adipocytes size and serum triglycerides levels, as well as target genes levels reduction in adipose tissue [113].

Although several studies showed successful results in animal models, there are still challenging issues to overcome in order to consider miRNAs as feasible therapeutic targets in insulin resistance. In particular, a critical point regards miRNA mimics because of their relative instability and alterations of their biological properties by chemical modification. For example, it will be important to achieve the ability to target miRNAs in selected cells and tissues involved in the specific disease of interest, considering also the potential off-targets effects caused by the pharmacological modulation of miRNAs expression (using miRNA mimics or miRNA inhibitors). Moreover, improvement of techniques for in-vivo specific-delivery of “therapeutic” miRNAs will be necessary, in order to elicit the exactly desired miRNA expression outcome, such as the restoration of physiological levels of a miRNA deregulated in a specific tissue. Finally, for the treatment of complex diseases, such as T2D, a multiple miRNAs combination therapy could be necessary to further improve therapeutic efficacy and success.

MiRNAs expression can also be indirectly modulated, for example by dietary components, as shown by the study of Parra et al. [157]. Briefly, expression of miR-143 (related to adipocyte differentiation), miR-103 and miR-107 (related to lipid metabolism) as well as of miR-221 and miR-222 (altered in obesity) was analyzed in retroperitoneal adipose tissue of mice fed with a standard or with a high fat diet and treated with conjugated linoleic acid. Results showed that miRNAs expression was influenced by conjugated linoleic acid treatment. Additionally, changes in miRNAs expression levels correlated with the expression of several adipocyte genes; specifically, miR-103 and miR-107 with genes involved in fatty acid metabolism, whereas miR-221 and miR-222 with the expression of adipo-cytokines. Joven et al. described that a continuous oral administration of high-dose plant-derived polyphenols in hyperlipidemic mice (C57BL/6J background mice deficient in LDL receptor) contributed to modulate hepatic expression of selected miRNAs. More in detail, a high-fat diet increased the expression of miR-103 and miR-107 (respect to a chow diet) and this effect was completely reversed by polyphenols. Dietary polyphenols also decreased the expression of miR-122, attenuated weight gain, liver steatosis and insulin resistance during fat-rich diet in animal models [158].

Collectively, these results demonstrate that miRNAs involved in insulin sensitivity and insulin signaling regulation can be directly or indirectly modulated in-vivo and may potentially represent candidate therapeutic targets for T2D insulin resistance.

## 8. Conclusions

Taken together, the entire set of studies reported in this review strengthens the role of miRNAs not only as regulators of glucose homeostasis through the specific modulation of insulin signaling components, but also as potential therapeutic targets and/or agents ameliorating insulin-resistance and other pathogenic factors contributing to T2D.

## Abbreviations

IRS1/2	insulin receptor substrate 1/2
PIP3	phosphatidylinositol 3,4,5-triphoshate
PIP2	phosphatidylinositol 4,5-bisphoshate
PTEN	Phosphatase and Tensin Homolog
SHIP2	SH2 Domain-Containing Inositol 5-Phosphatase 2
PDK1	phosphoinositide-dependent kinase 1
AKT	Protein Kinase B
FOXO1	forkhead box protein O1
GSK3β	glycogen synthase kinase 3 beta
mTOR	mammalian target of rapamicin
BCL2-	Bad associated agonist of Cell Death
PP2A	Protein phosphatase 2 A
PHLPP	PH domain leucine-rich repeat protein phosphatase
Cav-1	Caveolin 1
PTPN1	Protein Tyrosine Phosphatase Non-Receptor Type 1
PTP1B	Protein Tyrosine phosphatase 1B
Grb2	Growth factor Receptor-Bound protein 2
SOS	Son of Sevenless
KRAS	Kirsten rat sarcoma viral oncogene homolog;
RAF	RAF proto-oncogene serine/threonine-protein kinase
MEK	mitogen-activated protein kinase kinase
ERK	Extracellular signal-Regulated Kinase
HFD	High Fat Diet
DIO	Diet Induced Obesity
3’UTR	3’ Untranslated Region
GRB10	Growth Factor Receptor Bound Protein 10
PKB/AKT	Protein Kinase B
TNFα	Tumor Necrosis Factor-Alpha
PTEN	Phosphatase And Tensin Homolog
SHIP2	SH2 Domain-Containing Inositol Phosphatase 2
mTOR	Mammalian Target of Rapamicin
FOXOs	Forkhead box O
PKBβ	Protein Kinase B subunit ß
IGFBP	Insulin-like Growth Factor Binding Protein
CpG	--C--phosphate--G--
TFIIB	Transcription Factor II B
miR	microRNA
KO	Knock Out
HepG2	Hepatoma G2
TGFß	Transforming Growth Factor ß
SMAD3	Mothers Against Decapentaplegic Member 3
ob/ob	Obese
INSRβ	Insulin Receptor ß
p-AKT	phosphorylated AKT (Protein Kinase B)
NAFLD	Non-alcholic Fatty Liver Disease
NASH	Non-alcholic Steatohepatitis
PTPN1	Protein Tyrosine Phospatase, Non-receptor type 1
PTP1B	Protein-tyrosine phosphatase 1B
HNF4α	Hepatocyte Nuclear Factor 4 a
TNFα	Tumor Necrosis Factor a
PIK3R3	Phosphatidylinositol 3-kinase regulatory subunit gamma
SREBP-1	Sterol regulatory element-binding protein 1
SREBP-2	Sterol regulatory element-binding protein 2
ERK	Extracellular Signal-Regulated Kinase
db/db	Diabetic
FOXO-KO	Forkhead box O-Knock Out
p-FOXO1	Phosphorylated Forkhead box O 1
INS1-E	Insulinoma 1-E
PP2A	Protein Phosphatase 2 A
PHLPPs	Pleckstrin Homology Domain Leucine-rich Repeat Protein Phosphatases
MAPK	Mitogen-Activated Protein Kinase
VEGF	Vascular Endothelial Growth Factor
HIF-Iα	Hypoxia-inducible factor 1-a
RCC4	Renal cancer cell 4
IGFBP5	Insulin-like Growth Factor Binding Protein 5
PPARγ	Peroxisome Proliferator Activated Receptor γ
GalNAc	Galactose/N-acetylgalactosamine
SIRT1	Sirtuin 1
LDL	Low Density Lipoprotein
RNAse	Ribonuclease
Ran GTP	RAs-related Nuclear protein Guanosine tetraphosphate
